# Knockout of p21-Activated Kinase 4 Stimulates MHC I Expression of Pancreatic Cancer Cells via an Autophagy-Independent Pathway

**DOI:** 10.3390/cancers17030511

**Published:** 2025-02-03

**Authors:** Yi Ma, Chelsea Dumesny, Li Dong, Ching-Seng Ang, Mehrdad Nikfarjam, Hong He

**Affiliations:** 1 Department of Surgery, Austin Precinct, University of Melbourne, Heidelberg, VIC 3084, Australia; y.ma11@student.unimelb.edu.au (Y.M.); watsoncj@unimelb.edu.au (C.D.); li.dong@unimelb.edu.au (L.D.); m.nikfarjam@unimelb.edu.au (M.N.); 2 Department of General Surgery, Monash Health, Clayton, VIC 3806, Australia; 3 Mass Spectrometry and Proteomics Facility, Bio21 Molecular Science and Biotechnology Institute, University of Melbourne, Parkville, VIC 3052, Australia; ching-seng.ang@unimelb.edu.au; 4 Department of Hepato-Pancreato-Biliary Surgery, Austin Health, Heidelberg, VIC 3084, Australia

**Keywords:** p21-activated kinase 4, pancreatic cancer, MHC I, autophagy, chloroquine

## Abstract

Background/Objectives: Pancreatic ductal adenocarcinoma (PDA) is one of the most malignant solid cancers. KRAS mutation accounts for over 90% of cases. p21-activated kinases (PAKs) act downstream of KRAS and are involved in tumorigenesis. The inhibition of PAK4 suppresses PDA by stimulating the tumor infiltration of cytotoxic T cells. The major histocompatibility complex class I (MHC I) is a key in presenting antigens to cytotoxic T cells. MHC I degradation via autophagy promotes the immune evasion of pancreatic cancer. We investigated the effect of PAK4 inhibition on MHC I expression and autophagy. Methods: In this study, using proteomic analysis, fluorescence-activated cell sorting (FACS), and immunoblotting, we examined the effect of PAK4 knockout (KO) in human PDA cells on the expression of MHC I and autophagy to identify the mechanism involved in the stimulation of cytotoxic T cells by PAK4 inhibition. Results: We found that PAK4 KO increased MHC I expression in two human PDA cell lines: MiaPaCa-2 and PANC-1. PAK4 KO also increased cancer cell autophagy. However, the inhibition of autophagy by chloroquine (CQ) did not affect the effect of PAK4 KO on apoptosis and cell death. More importantly, the inhibition of autophagy by CQ did not alter the expression of MHC I stimulated by PAK4 KO, indicating that PAK4 KO stimulated MHC I expression via an autophagy-independent pathway. Conclusions: We identified a role of PAK4 in MHC I expression by PDA cells, which is independent of autophagy.

## Simple Summary

KRAS mutations occur in over 90% of pancreatic ductal adenocarcinoma (PDA) cases. p21-activated kinases (PAKs) act downstream of KRAS and are involved in tumorigenesis. The inhibition of PAK4 suppresses PDA by stimulating cytotoxic T cells. The major histocompatibility complex class I (MHC I) is a key in presenting antigens to cytotoxic T cells. MHC I degradation via autophagy promotes the immune evasion of PDA. We investigated the effect of PAK4 on the MHC I expression of PDA cells and its relation to autophagy to reveal the mechanism(s) involved in anti-tumor immunity stimulated by PAK4 inhibition. Our findings advance the knowledge of the tumor immune response promoting PDA immunotherapy.

## 1. Introduction

Pancreatic ductal adenocarcinoma (PDA) is one of the most malignant solid cancers, with a dismal survival prognosis of below 10% within 5 years [1]. Surgery remains the only cure for PDA, and systemic therapy only extends survival by less than 12 months [2,3,4]. KRAS mutation accounts for over 90% of PDA cases [5], and p21-activated kinases (PAKs) act downstream of KRAS. Among the six members of the PAK family, PAK4 has attracted increasing recognition for its role in pancreatic cancer tumorigenesis. PAK4 plays a role in PDA cell proliferation, apoptosis, invasion, and chemoresistance [6,7,8,9,10,11].

Recent studies have demonstrated that the inhibition of PAK4 increased CD8+ T cell infiltration in immune-resistant melanoma and prostate cancer and synergized with immune checkpoint inhibitors in suppressing cancer growth [12,13,14]. Inhibition of PAK4 increased the MHC I expression in oral squamous cell carcinoma but not in melanoma [15]. PAK4 knockout (KO) stimulated the CD8+ T-cell response in PDA [16]. The inhibition of PAK4 contributed to the normalization of the vasculature, promoting T-cell infiltration to inhibit glioblastoma growth in mice [17]. However, the mechanisms involved in the stimulation of CD8+ T cells via the inhibition of PAK4 are not clear. Additionally, the effect of PAK4 inhibition on the expression of immune markers including MHC I on PDA cell surfaces has not been examined [12,14]. The antigen presentation of MHC I is critical for activating the tumor-killing CD8+ T -cell immune response. The low expression of MHC I leads to reduced immunogenicity of a tumor, contributing to tumour immune escape and to immunotherapy resistance [18].

Autophagy of PDA cells degraded the cell surface’s MHC-I complex, which protected cancer cells from being killed by cytotoxic CD8+ T cells [19]. The inhibition of autophagy by chloroquine (CQ), a clinically available antimalarial agent that inhibits the acidification of the lysosomes and autophagy, increased the cell surface expression of MHC I on murine PDA cells in vitro and in vivo as well as sensitized the PDA response to dual immune checkpoint inhibitors, associated with the increased infiltration of cytotoxic T cells [19,20]. Furthermore, PAK4 knockdown (KD) in the human hepatocellular carcinoma (HCC) HepG2 cell line induced autophagy and caused G2/M cell-cycle arrest [21]. This is consistent with the observed upregulation of sequestosome 1 (SQSTM, a known cargo receptor in autophagy [22]) in the proteomic results from cells treated with a novel PAK4 inhibitor, PAKib [23]. However, how PAK4-associated autophagy affects PDA cell survival and immune response remains unknown, given autophagy can play different roles in cancer cells [24].

In the current study, we determined the effect of PAK4 KO on the expression of immune markers, especially MHC I by human PDA cells, and autophagy. We also assessed the biological effects of the inhibition of autophagy on PAK4 KO cancer cells, especially on the expression of cell surface immune markers such as MHC I.

## 2. Materials and Methods

### 2.1. Generate PAK4 Knockout Cells

The PAK4 KO PDA cell lines were generated using an inducible lentiviral CRISPR/Cas9 system as described previously [25]. The pFgH1tUTG GFP lentiviral vector carrying single-guide RNAs targeting human PAK4 (guide 1: GCAGCCGAGGCCGGTTCGC; guide 2: GCTTCGACCAGCACGAGCAG) were transfected to MiaPaCa-2 and PANC-1 cells. The single clones of transfected cells were selected using BD FACS Aria III (BD Biosciences, Jersey City, NJ, USA). PAK4 KO expression was determined by immunoblotting. The PAK4 KO CRISPR event was determined using primers listed in Table S1 and analyzed using the method provided in the Supplementary Method. The PAK4 KO CRISPR event was confirmed as shown in Figure S1.

### 2.2. Cell Proliferation Assay

The human pancreatic cancer cell lines MiaPaCa-2 and PANC-1 were purchased from the American Type Culture Collection and cultured in Dulbecco’s modified Eagle’s medium (DMEM) (Hyclone Laboratories, Melbourne, VIC, Australia) supplemented with 5% fetal bovine serum (FBS) (Hyclone Laboratories, VIC, Australia). Wild-type (WT) and PAK4 KO MiaPaCa-2 as well as PANC-1 cells (5000 cells/well) were incubated for 4, 24, 48, and 72 h. For chloroquine diphosphate (CQ) (Sigma-Aldrich, St. Louis, MO, USA) treatment, the cells were incubated in DMEM with 5% FBS overnight and then incubated with or without CQ in DMEM for 24 h. The cell proliferation was determined by MTT assays.

### 2.3. Immunoblot

WT and PAK4 KO cells were cultured with or without 20 μM CQ for 24 h and were then lysed with ONYX buffer (Table S2) with phosphatase inhibitor (Roche, Mannheim, Germany) and protease inhibitor (Roche). Protein concentration was quantified by DC protein assays (Bio-Rad, Hercules, CA, USA). Proteins were separated in 10% sodium dodecyl-sulfate (SDS) gel and then transferred onto a nitrocellulose membrane, followed by blotting with primary antibodies against PAK4, LC3B, ATG5, Beclin 1, SQSTM1, BCL2, and GAPDH (Table S3). After incubation with goat anti-rabbit IgG(H+L)-HRP conjugate (Bio-Rad), the protein blots were detected in ECL SelectTM Detection Reagent (Cytiva, Amersham Place, UK) and captured using a ChemiDocTM MP Imaging system (Bio-Rad). The density of each blot was analyzed using ImageJ 2 [26].

### 2.4. Flow Cytometry Analysis

#### 2.4.1. Monodansylcadaverine Staining

WT and PAK4KO MiaPaCa-2 and PANC-1 cells (1 × 10^5^ cells/well) were cultured for 24 h and then incubated with monodansylcadaverine (MDC) dye (Sigma-Aldrich) at 37 °C for 15 min. Cells were trypsinized, collected, and incubated with 10 μg/mL Propidium Iodide (PI) (Sigma-Aldrich, St. Louis, MO, USA) at 4 °C in the dark and then subjected to a FACSymphony A3 flow cytometer (BD Biosciences). The background autofluorescence of the WT and PAK4 KO MiaPaCa-2 as well as PANC-1 cells was also determined after spectral compensation. The adjusted median fluorescence intensity (MFI) was calculated by subtracting the MFI of cell-line-specific autofluorescence from the individual immune marker, MFI, of the study sample (Figure S2). Manual gating and statistical analysis were performed using FCS Express version 7.12.0007 (De Novo Software, Pasadena, CA, USA).

#### 2.4.2. Determine the Expression of Cell Surface Immune Markers

WT and PAK4KO MiaPaCa-2 and PANC-1 cells (1 × 10^5^ cells/well) were incubated for 24 h, with or without Interferon-gamma (IFN-γ, 24 ng/mL) or 20 μM CQ. Cells were then trypsinized and collected in FACS buffer (Table S2). Zombie UVTM fixable viability dye (BioLegend, CA, USA) was used to determine cell viability.

Antibodies against human major histocompatibility complex I (MHC I), major histocompatibility complex II (MHC II), and programmed death-ligand 1 (PDL1) (Table S4) were added and incubated on ice for 20 min in the dark. Cells were resuspended in FACS buffer and subjected to FACS analysis using a Cytek^®^ Aurora flow cytometer (Cytek Biosciences, Fremont, CA, USA). Manual gating and statistical analysis were performed using FCS Express version 7.12.0007.

### 2.5. Measure Apoptosis, Cell Death, and Cell Cycle Analysis

WT and PAK4 KO MiaPaCa-2 as well as PANC-1 cells (1 × 10^5^ cells/well) were incubated with or without CQ (20 μM) CQ for 24 h before being subjected to FACS. The apoptosis, cell death, and cell cycle analyses were performed using previously described methods [16]. An APC Annexin V apoptosis detection kit (BioLegend) and a Click-iTTM Plus EdU Alexa FluorTM 647 flow cytometry assay kit (Invitrogen, Waltham, MA, USA) were used for apoptosis/death and cell cycle analysis, respectively.

### 2.6. Proteomic Analysis

The proteomic analysis was conducted as previously described [16]. The procedure included sample preparation, liquid chromatograph data-independent acquisition mass spectrometry, database search, and bioinformatic analysis. For sample preparation, WT and PAK4 KO MiaPaCa-2 cells were seeded in a 10 cm culture dish, cultured until 80% confluence, and then lysed with radioimmunoprecipitation assay (RIPA) buffer (for details, see Table S2) with protease and phosphatase inhibitors, followed by acetone precipitation and digestion to peptides for liquid chromatograph data-independent acquisition with mass spectroscopy a subsequent data search, which were performed according to the protocol described before [16]. The bioinformatic analysis was conducted following previously reported methods [16]. The gene set enrichment analysis (GSEA) of significant proteins was conducted based on Gene Ontology Biological Process (GOBP) annotations [27]. The functional enrichment analysis of significant proteins was also conducted using the STRING database, version 12.0, and protein–protein interaction (PPI) networks were constructed based on the functional enrichment results [28].

Proteomic data were analyzed with R Statistical Software version 4.3.0 (R Core Team, 2021) [29]. The protein–protein interaction (PPI) network was constructed with stringApp version under 2.0.1 Cytoscape version 3.10.0 [30,31].

### 2.7. Statistical Analysis

The data were obtained from in vitro experiments conducted in three replicates. For continuous variables, mean ± standard deviation (SD) is reported for parametric data, while median ± inter-quantile range (IQR) is reported for non-parametric data. A two-sided t-test or one-way ANOVA was used for parametric data, Mann–Whitney’s U test for non-parametric data, and chi-square test for categorical data. Cell proliferation was analyzed using a linear regression model, and correlation coefficients were compared between groups. GraphPad Prism version 10.0.2 (GraphPad Software, Boston, MA, USA) was used for statistical analysis. *p*-value < 0.05 was considered statistically significant.

## 3. Results

### 3.1. PAK4 Knockout Differentially Affected the Growth of Pancreatic Cancer Cells

**Figure 1:**
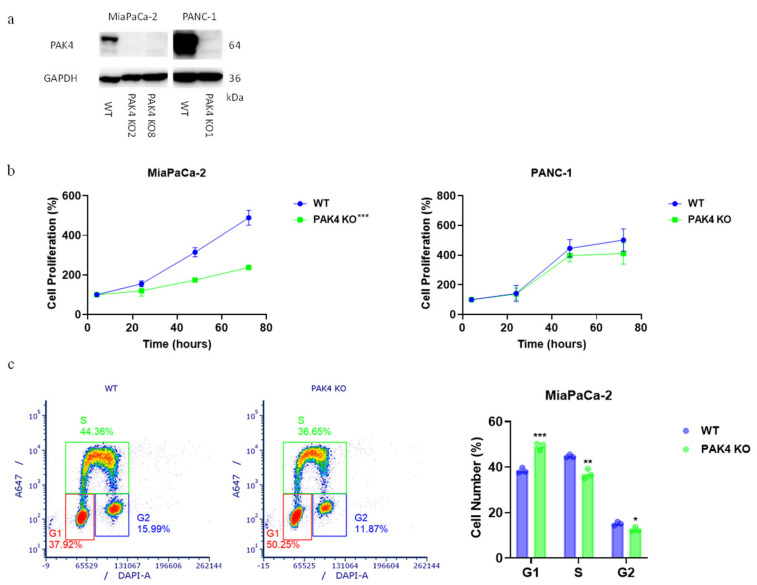
PAK4 knockout reduced MiaPaCa-2 cancer cell growth via G1/S cell cycle arrest. Expression of PAK4 in wild-type (WT) and PAK4 knockout (KO) MiaPaCa-2 as well as PANC-1 cell lines (**a**) were determined by immunoblotting. PAK4 KO reduced proliferation of MiaPaCa-2 but not PANC-1 cell lines in MTT assay. (**b**) Readouts of each cell lines at 4 h were taken as 100%. PAK4 KO induced G1/S cell cycle arrest in MiaPaCa-2 cell line (**c**). * *p* < 0.05, ** *p* < 0.01, *** *p* < 0.001, compared to WT, unless otherwise indicated.

The *PAK4* gene was knocked out in human PDA MiaPaCa-2 and PANC-1 cell lines using the CRISPR-CAS9 technique. The clones lacking PAK4 protein expression was confirmed using immunoblotting (Figure 1a). PAK4 knockout (KO) suppressed the cancer cell proliferation of MiaPaCa-2 cells (MiaPaCa-2 PAK4 KO8 was used in all assays) but not of PANC-1 cells (Figure 1b). The effect of PAK4 KO on MiaPaCa-2 cell proliferation was further evaluated using EdU staining. PAK4 KO increased the cell number in the G1 phase while reducing the percentage of cells in the S and G2 phases, suggesting that the reduced cell proliferation was due to G1/S cell cycle arrest.

### 3.2. PAK4 Knockout Stimulated the Expression of MHC I in Pancreatic Cancer Cells

**Figure 2:**
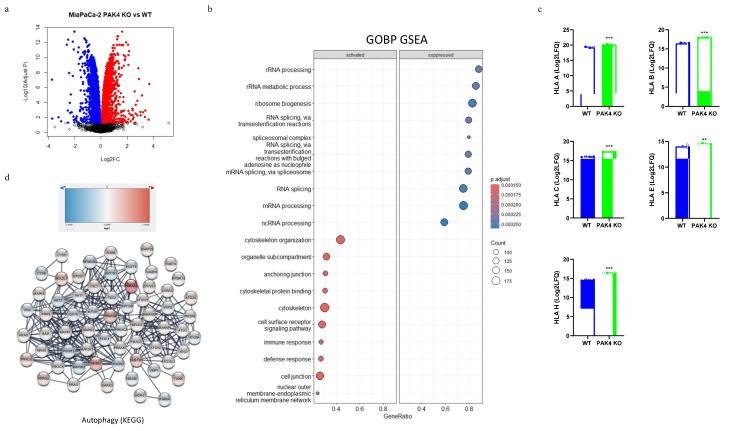
PAK4 knockout promoted MHC I expression and induced changes in autophagy. The cell lysates of wild-type (WT) and PAK4 knockout (KO) MiaPaCa-2 cells were subjected to proteomic analysis. The differential expressions of the protein profiles between WT and PAK4 KO cells are demonstrated using a volcano plot (**a**). Red colour indicates up-regulation of expression, while blue colour represents down-regulation of expression. The gene set enrichment analysis (GSEA) of GO biological process (GOBP) terms suggested an upregulation of the immune response in PAK4 KO MiaPaCa-2 cells (**b**). The expressions of HLA A, HLA B, HLA C, HLA E, and HLA H in PAK4 KO cells were significantly higher than in WT cells (**c**). Protein–protein interaction network analysis showed an enrichment of the KEGG autophagy pathway (**d**). HLA: human leukocyte antigen; ** *p* < 0.01, *** *p* < 0.001, compared to WT unless otherwise indicated.

We and others have demonstrated that the inhibition of PAK4 via knockout or small-molecular inhibitors, stimulates the cytotoxic T cells in tumor tissues to enhance the antitumor immunity [13,16]. MHC molecules are responsible for presenting an antigen to cytotoxic T cells, triggering an immune response and therefore may mediate the stimulatory effect of PAK4 KO on cytotoxic T cells. The data from the analysis of a global proteome showed the differential expressions of protein profiles between the WT and PAK4 KO (Figure 2a). Gene set enrichment analysis (GSEA) suggested that PAK4 KO not only suppressed RNA processing in MiaPaCa-2 cells but might have also activated the immune response (Figure 2b). A more in-depth examination of the upregulated proteins showed that PAK4 KO increased the expressions of human leukocyte antigens (HLAs) A, B, C, E, and H, which are all MHC I antigens (Figure 2c). Furthermore, the data from the functional enrichment analysis showed an upregulation of the autophagy pathway, signified by the increased expression of cargo receptor SQSTM1 and ATG8 family proteins (GABARAP and GABARAPL1) (Figure 2d), suggesting that PAK4 KO may increase MHC I expression via the regulation of autophagy, as a recent study indicated a potential connection between pancreatic cancer cell autophagy and cell surface MHC I expression [19].

To validate the results from the proteomic study, we determined the expression profile of MHC I, MHC II, and PDL1 in the WT and PAK4 KO MiaPaCa-2 cells and PANC-1 cells. IFN-γ was used as a positive control as IFN-γ is known to increase the cancer cell surface expressions of MHC I and PDL1 [32]. As shown in Figure S3, IFN-γ treatment increased the cell surface MHC I, MHC II, and PDL1 expressions of MiaPaCa-2 cells but not of PANC-1 cells. This suggests heterogeneity in the immune response among human PDA cells.

**Figure 3:**
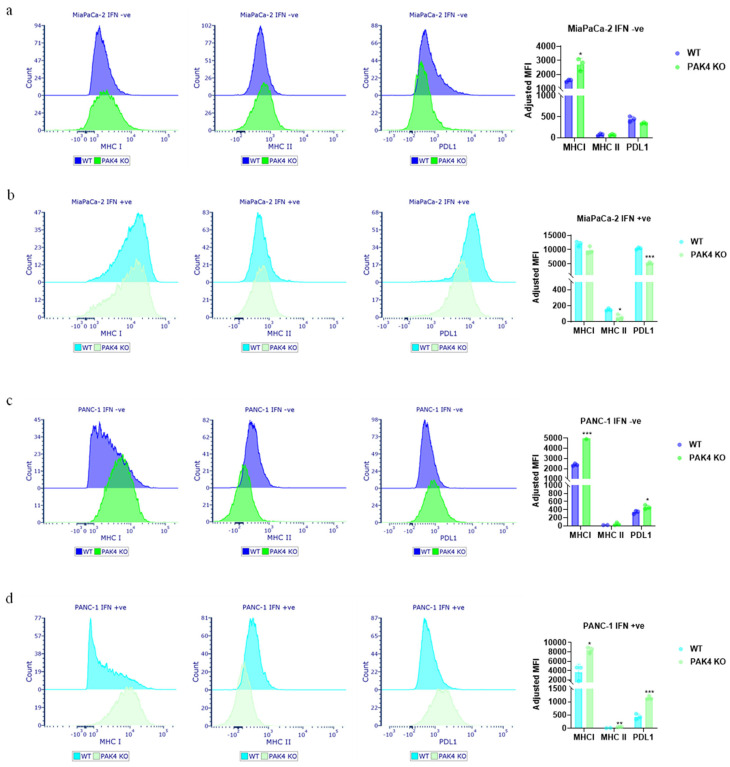
PAK4 knockout stimulated the cell surface expression of MHC I of MiaPaCa-2 and PANC-1 cells. The cell surface expressions of MHC I, MHC II, and PDL1 were determined by FACS analysis. In the absence of IFN-γ, PAK4 knockout (KO) stimulated the expression of MHC I of MiaPaCa-2 cells (**a**) but did not affect the expressions of MHC II and PDL1. In the presence of IFN-γ, PAK4 KO decreased the expressions of MHC II and PDL1 of MiaPaCa-2 cells (**b**) but did not affect the expression of MHC I. PAK4 KO increased the expressions of MHC I, MHC II, and PDL1 of PANC-1 cells in the absence (**c**) and presence (**d**) of IFN-γ. * *p* < 0.05, ** *p* < 0.01, *** *p* < 0.001, compared to WT, unless otherwise indicated.

Furthermore, the expressions of cell surface MHC I, MHC II, and PDL1 in the presence and absence of IFN-γ were compared between WT and PAK4 KO MiaPaCa-2 as well as PANC-1 cells. PAK4 KO increased the cell surface MHC I expression on MiaPaca-2 and PANC-1 cells (Figure 3a,c) without IFN-γ. However, while PAK4 KO did not affect the cell surface MHC II and PDL1 expressions in MiaPaCa-2 cells, PAK4 KO increased the PDL1 level in PANC-1 cells. In the presence of IFN-γ, PAK4 KO did not increase the MHC I level further in MiaPaCa-2 cells but reduced the MHC II and PDL1 levels (Figure 3b). This was likely due to the saturation of MHC I expression of MiaPaCa-2 cells in response to IFN-γ treatment. On the other hand, PAK4 KO increased the expressions of MHC I and PDL1 in PANC-1 cells regardless of IFN-γ (Figure 3d), as PANC-1 cells are resistant to IFN-γ (Figure S3). The expression of MHC II was low in both MiaPaCa-2 and PANC-1 cells with or without IFN-γ, suggesting the lack of an antigen-presenting cell (APC) phenotype in these PDA cells. These results indicated that PAK4 KO stimulated MHC I expression in human PDA cells, while its effect on PDL1 expression was cell specific.

### 3.3. PAK4 Knockout Differentially Regulated the Autophagy of Pancreatic Cancer Cells

As a previous study reported that the inhibition of autophagy contributed to increased MHC I expression, we assessed the effect of PAK4 KO on PDA cell autophagy [19].

**Figure 4:**
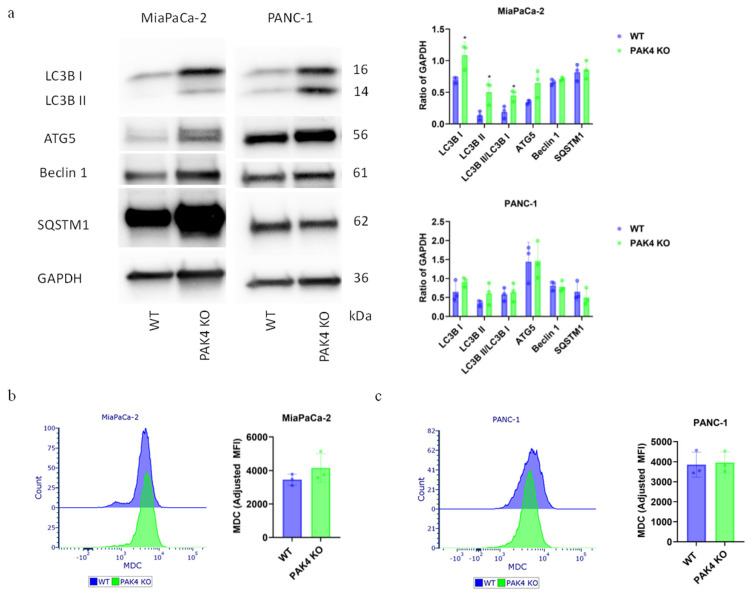
PAK4 knockout induced changes in autophagy of MiaPaCa-2 and PANC-1 cells. PAK4 knockout (KO) induced changes in the expressions of LC3B, ATG5, Beclin 1, and SQSTM1 in WT versus PAK4 KO MiaPaCa-2 and PANC-1 cells. (**a**). FACS analysis of monodansylcadaverine (MDC) staining in WT versus PAK4 KO of MiaPaCa-2 cells (**b**) or PANC-1 cells (**c**). * *p* < 0.05, compared to WT, unless otherwise indicated.

PAK4 KO significantly increased LC3B expression and the conversion of LC3B I to LC3B II in the MiaPaCa-2 cell line but not in the PANC-1 cell line (Figure 4a). This suggested that PAK4 KO caused a greater induction of autophagy in MiaPaCa-2 cells in comparison to PANC-1 cells. However, PAK4 KO did not alter the levels of other autophagy markers such as ATG5, Beclin1, and SQSTM1 in either cell line (Figure 4a). As international guidelines recommended using more than one method to confirm autophagy level changes, we also stained the cells with MDC and measured its level with FACS [33,34]. MDC is a dye known to accumulate in autophagic vesicles and emits blue fluorescence [35]. However, PAK4KO failed to significantly increase the MDC staining of either MiaPaCa-2 cells (Figure 4b) or PANC-1 cells (Figure 4c). Together, these results suggested that PAK4 KO induced a stronger response in the autophagy of MiaPaCa-2 cells than of PANC-1 cells.

### 3.4. Inhibition of Autophagy by Chloroquine Did Not Change the Effect of PAK4 KO on Cancer Cell Growth

**Figure 5:**
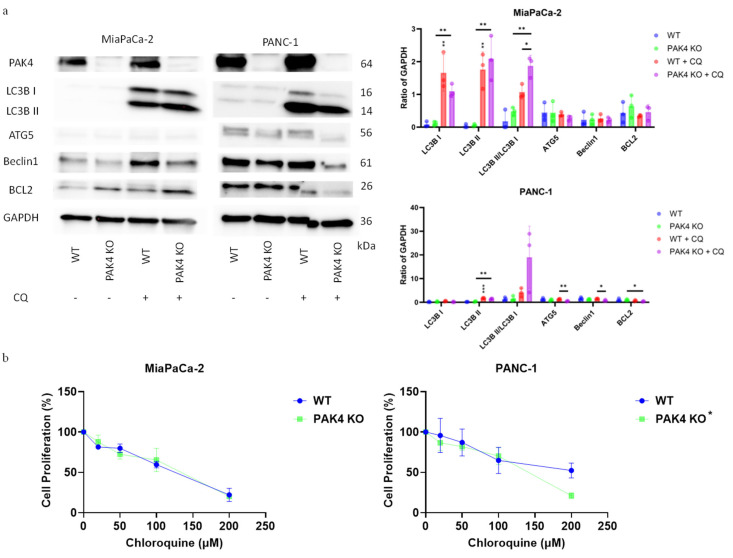
The effect on inhibition of autophagy by chloroquine in PAK4 knockout MiaPaCa-2 and PANC-1 cells. (**a**) PAK4 knockout (KO)-induced changes in the expressions of LC3B, ATG5, Beclin 1 and SQSTM1 with or without chloroquine (CQ). (**b**) Cell proliferation of wild-type (WT) versus PAK4 KO MiaPaCa-2 and PANC-1 cells treated with increasing concentrations of chloroquine per MTT assay. * *p* < 0.05, ** *p* < 0.01, *** *p* < 0.001, compared to WT, unless otherwise indicated.

Chloroquine (CQ) is known as an anti-malarial agent but can also inhibit autophagy by neutralizing lysosomal acidity and thus prevent autophagosome–lysosome fusion [20]. CQ treatment resulted in much greater increases in LC3B II expression and the LC3B II/LC3B I ratio in the WT and PAK4 KO MiaPaCa-2 as well as PANC-1 cells (Figure 5a). However, while CQ did not significantly affect the levels of ATG5 and Beclin1 in MiaPaCa-2 cells, it reduced the ATG5 and Beclin1 expressions in PANC-1 PAK4 KO cells (Figure 5a). The interaction between Beclin1 and BCL2 is known to induce apoptosis by releasing pro-apoptotic BAX/BAK [36]. CQ did not significantly affect the BCL2 level in PAK4 KO MiaPaCa-2 cells but reduced the BCL2 expression in PAK4 KO PANC-1 cells, suggesting a potential effect on apoptosis (Figure 5a). These results confirmed that PAK4 KO differentially induced autophagy in these cell lines.

As autophagy is recognized for its cytoprotective effect on cancer cell survival, we assessed the combined effect of CQ and PAK4 KO on MiaPaCa-2 and PANC-1 cell growth [22]. CQ dose-dependently inhibited the cell growth of both the WT and PAK4 KO of either MiaPaCa-2 or PANC-1 cells (Figure 5b). CQ did not show a greater inhibitory effect on PAK4 KO MiaPaCa-2 and PANC-1 cells at low concentrations (Figure 5b). However, it did suppress PANC-1 PAK4 KO cells more significantly at high concentrations, but this is unlikely to have in vivo meaning due to the high toxicity of CQ (Figure 5b).

Given the changes in BCL2, we also evaluated the effect of CQ on WT and PAK4 KO cancer cell apoptosis. PAK4 KO protected both MiaPaCa-2 and PANC-1 cells from cell death but only reduced apoptosis in MiaPaCa-2 cells (Figure S4). CQ did not affect the apoptosis of either cell line but increased cell death in both cell lines (Figure S4a,b). PAK4 KO also protected against cell death in both cell lines in the presence of CQ (Figure S4a,b). These results indicated that CQ did not reverse the cytoprotective effect of PAK4 KO, while CQ inhibited autophagy in PDA cell lines and induced cell death.

### 3.5. Inhibition of Autophagy by Chloroquine Did Not Block the PAK4 KO-Stimulated Expression of MHC I in Pancreatic Cancer Cells

**Figure 6:**
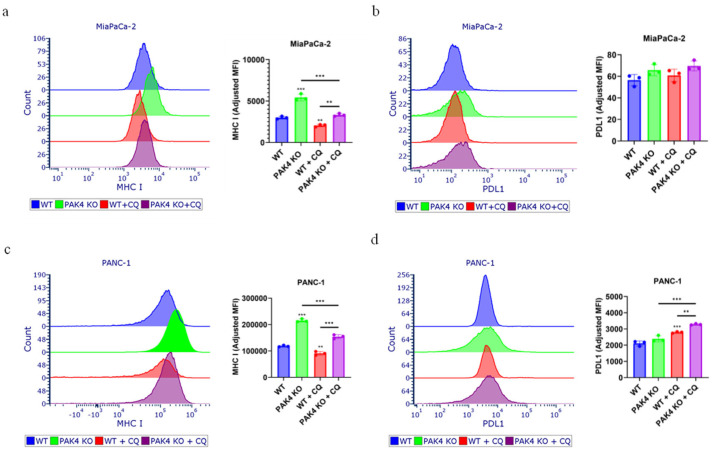
PAK4-knockout-stimulated expression of MHC I was not blocked by inhibition of autophagy by chloroquine. Cell surface expressions of MHC I (**a**,**c**) and PDL1 (**b**,**d**) were determined by FACS analysis. PAK4 knockout (KO) increased expression of MHC I in MiaPaCa-2 (**a**) and PANC-1 (**c**) with or without chloroquine (CQ). PAK4 KO did change expression of PDL1 in MiaPaCa-2 cells treated with or without CQ (**b**). Expression of PDL1 in PANC-1 was not changed by PAK4 KO without CQ but increased in presence of CQ (**d**). ** *p* < 0.01, *** *p* < 0.001, compared to WT, unless otherwise indicated.

The above results demonstrated that PAK4 KO stimulated MHC I expression in both MiaPaCa-2 and PANC-1 cells (Figure 2) and that PAK4 KO differentially affected autophagy (Figure 4). To determine whether PAK4 KO-stimulated MHC I expression is autophagy-dependent or not, the expression of MCH I of the WT and PAK4 KO cells was measured in the presence of CQ. PAK4 KO increased the MHC I expression of both MiaPaCa-2 and PANC-1 cell lines in the absence and presence of CQ (Figure 6a,c), indicating that the inhibition of autophagy by CQ did not block the increased expression of MHC I by PAK4 KO, which suggested that PAK4 KO-stimulated MHC I expression was independent of autophagy. CQ suppressed the cell surface MHC I expression of both MiaPaCa-2 and PANC-1 cells (Figure 6a,c). In addition to MHC I, the effect of CQ on PDL1 expression was assessed given that both MiaPaCa-2 and PANC-1 cells showed high PDL1 expression (Figure 3). While neither PAK4 KO nor CQ had any effect on MiaPaCa-2 cell surface PDL1 expression, CQ increased the PDL1 expression of PANC-1 cells and more so in PAK4 KO PANC-1 cells (Figure 6b,d). These results suggested that PAK4 KO stimulated the expression of MHC I by pancreatic cancer cells via autophagy-independent pathway and that PAK4 KO differentially affected the expression of PDL1 of pancreatic cancer cells.

## 4. Discussion

The role of PAK in tumorigenesis has been reported extensively [22]. Recent emerging evidence has also pointed to a role of PAK4 in cancer immune evasion in melanoma, prostate cancer, and pancreatic cancer [13,14,16]. The inhibition of PAK4 suppressed tumor growth by stimulating the infiltration of cytotoxic T cells and by sensitizing the tumor response to immune checkpoint inhibitors and to CAR-T immunotherapy [13,14,17]. However, the mechanism involved is not well understood, although previous studies have suggested that the inhibition of PAK4 reprograms the tumor vasculature to promote the infiltration of T cells [12,14,17]. We thought that PAK4 inhibition could affect the expression of immune markers, especially MHC I, on the cancer cell surface to stimulate T-cell infiltration. Indeed, for the first time, we demonstrated that PAK4 KO increased the expression of MHC I by PDA cells and that the inhibition of autophagy did not affect this PAK4-KO-stimulated expression of MHC I, indicating that PAK4 KO stimulated the expression MHC I, possibly via an autophagy-independent pathway.

MHC molecules are well known for their importance in antigen presentation and the activation of both CD4+ and CD8+ T cells [37]. While MHC I is ubiquitously expressed by all nucleated cells, MHC II is traditionally believed to be solely expressed by professional antigen-presenting cells (APCs) [38]. However, MHC II molecules were recently found to be present on PDA cancer cells and can lead to CD4+ T-cell killing of cancer cells [39]. In this study, we reported an increase in MHC I expression by PAK4 KO in two human PDA cell lines, which would at least be partially responsible for the increased cytotoxic T-cell infiltration by PAK4 inhibition in PDA. This also suggests that the effect of PAK4 inhibition on cancer cell surface MHC I expression is likely cancer-specific, given that it has been observed in PDA and oral SCC but not in melanoma [12,15]. Furthermore, given that PAKs are downstream players of RAS, this may also partially explain the effect of KRAS mutation on downregulating cancer cell surface MHC I [40]. On the other hand, the MHC II expression on PDA cancer cells was minimal in our analysis, which is unlikely to play a significant role in immune response. PDL1 is a known immune checkpoint molecule that inactivates the CD8+ T-cell response by coupling with PD1 on the T-cell surface [41]. While our results suggested an increased expression of PDL1 by PAK4 inhibition in the PANC-1 cell line, this was not the case for MiaPaCa-2. This indicates that PAK4 inhibition is likely to have a cell-specific effect on PDL1 expression due to cancer cell heterogeneity. The difference in the genetic profiles between MiaPaCa-2 and PANC-1 may contribute to the cell-line-specific response observed here. MiaPaCa-2 carries a mutation in codon 12 (G12C) of KRAS without mutations in SMAD4 or TP53, while PANC-1 has mutations in KRAS (G12D) and TP53 (P72R and R273H) [42].

Recently, PAK4 inhibition was found to induce autophagy in a human hepatocellular carcinoma (HCC) cell line causing G2/M cell cycle arrest and reducing cancer cell proliferation [21]. Given that cancer cell autophagy was also found to degrade cell surface MHC I in PDA [19], we further assessed the effect of PAK4 inhibition on autophagy, and its relation to the expressions of MHC I, MHC II, and PDL1 in human PDA cell lines. PAK4 inhibition induced changes in autophagy, predominantly in MiaPaCa-2 cells rather than in PANC-1 cells. However, these changes in autophagy did not affect the cell apoptosis or death of either human PDA cell line (Figure S4). The inhibition of autophagy by CQ did not change the trends in apoptosis, cell death, and proliferation (Figure S4 and Figure 5). More importantly, the increased expression of MHC I by PAK4 KO was not affected by the inhibition of autophagy, suggesting an autophagy-independent pathway involved in the PAK4-KO-stimulated expression of MHC I. As autophagy is a complex cellular mechanism and can play a variety of roles in cancer cell function, further investigation is required to determine the role of autophagy induced by PAK4 inhibition in PDA.

Our recent study showed an upregulation of cytotoxic CD8+ T-cell infiltration by PAK4 KO PDA cells, which led to tumor regression in murine syngeneic PDA models. The current finding that PAK4 KO increased cancer cell surface MHC I expression may serve as a potential mechanism for the induction of T-cell infiltration. However, to directly examine the activation of CD8+ T cells by PAK4 KO tumor cells, a co-culture study of human CD8+ T cells with PAK4 KO human PDA cells will be required. Furthermore, our current studies, together with previous evidence of PAK-KO-induced CD8+ T-cell infiltration in PDA, make PAK4 an important target for future treatment development. However, the development of PAK4 inhibitors has been challenged by their poor selectivity, which has resulted in their failure in phase 1 trials [22]. Our recent attempts to develop selective PAK4 inhibitors have also been limited by their poor solubility [23]. In addition, the rapid development of resistance to PAK4 inhibition by cancer cells remains another challenge to overcome [16]. The recent development of a PAK4-targeted PROTAC degrader may provide hope in the field, but its in vivo effect has not been assessed and thus will require further evidence to support its use [43].

## 5. Conclusions

We identified a role of PAK4 in MHC I expression in human PDA that is independent of autophagy. PAK4-KO-induced changes in autophagy did not affect the apoptosis, death, and proliferation of PDA cells nor the expression of MHC I. Our finding that PAK4 KO increased the expression of MHC I by PDA cells warrants further study of whether the increased expression of MHC I translates into more efficient cancer cell killing by CD8+ T cells.

## Data Availability

Data and study material will be made available upon request from the corresponding author.

## Supplementary Materials

The following supporting information can be downloaded at: https://www.mdpi.com/article/10.3390/cancers17030511/s1, Supplementary Method. Analysis of PAK4 knockout CRISPR event. Figure S1. Sequence analysis of PAK4KO CRISPR events. Figure S2. Unstained WT and PAK4 KO controls for cell surface MHC I, MHC II, and PDL1 FACS analysis after compensation. Figure S3. IFN-g induced MHC I and PDL1 expression in MiaPaCa-2 and PANC-1 cell lines. Figure S4. Inhibition of autophagy by chloroquine did not affect the effect of PAK4 knockout on apoptosis and cell death. Table S1, Primers for PAK4 CRISPR events. Table S2. Buffers used in the method. Table S3. Primary antibodies for immunoblotting. Table S4. Primary antibodies for flow cytometry.

## Abbreviations

APC	Antigen-presenting cell
ATG	Autophagy-related gene
BAK	BCL2 homologous antagonist/Killer
BAX	BCL2-associated protein X
BCL2	B-cell leukemia/lymphoma 2 protein
CAR	Chimeric antigen receptor
CAS	CRISPR-associated protein
CQ	Chloroquine
CRISPR	Clustered Regularly Interspaced Short Palindromic Repeat
DMEM	Dulbecco’s modified Eagle’s medium
FACS	Fluorescence-activated cell sorting
FBS	Fetal bovine serum
GABARAP	Gamma-aminobutyric acid receptor-associated protein
GAPDH	Glyceraldehyde-3-phosphate dehydrogenase
GOBP	Gene Ontology biological process
GSEA	Gene set enrichment analysis
HCC	Hepatocellular carcinoma
HLA	Human leukocyte antigen
HRP	Horseradish peroxidase
IFN	Interferon
IQR	Interquantile range
KD	Knockdown
KO	Knockout
KRAS	Kirsten rat sarcoma virus
LC3B	Microtubule-associated protein 1 light chain 3 beta
MDC	Monodansylcadaverine
MFI	Median fluorescence intensity
MHC	Major histocompatibility complex
MTT	Thiazolyl blue tetrazolium bromide
PAK	p21-activated kinase
PC	Pancreatic Cancer
PDA	Pancreatic ductal adenocarcinoma
PDL1	Programmed death ligand 1
PI	Propidium iodide
PPI	Protein–protein interaction
RIPA	Radioimmunoprecipitation assay
SD	Standard deviation
SDS	Sodium dodecyl-sulfate
SMAD	Suppressor of mothers against decapentaplegic
SQSTM1	Sequestosome 1
TP53	Tumor protein p53
WT	Wild-type

## References

[1] AIHW (2019). Cancer in Australia 2019.

[2] Burris H.A., Moore M.J., Andersen J., Green M.R., Rothenberg M.L., Modiano M.R., Cripps M.C., Portenoy R.K., Storniolo A.M., Tarassoff P. (1997). Improvements in survival and clinical benefit with gemcitabine as first-line therapy for patients with advanced pancreas cancer: A randomized trial. J. Clin. Oncol..

[3] Von Hoff D.D., Ervin T., Arena F.P., Chiorean E.G., Infante J., Moore M., Seay T., Tjulandin S.A., Ma W.W., Saleh M.N. (2013). Increased survival in pancreatic cancer with nab-paclitaxel plus gemcitabine. N. Engl. J. Med..

[4] Conroy T., Desseigne F., Ychou M., Bouche O., Guimbaud R., Becouarn Y., Adenis A., Raoul J.L., Gourgou-Bourgade S., de la Fouchardiere C. (2011). FOLFIRINOX versus gemcitabine for metastatic pancreatic cancer. N. Engl. J. Med..

[5] Maitra A., Hruban R.H. (2008). Pancreatic cancer. Annu. Rev. Pathol..

[6] Tyagi N., Bhardwaj A., Singh A.P., McClellan S., Carter J.E., Singh S. (2014). p-21 activated kinase 4 promotes proliferation and survival of pancreatic cancer cells through AKT- and ERK-dependent activation of NF-kappaB pathway. Oncotarget.

[7] Mohammad R.M., Li Y., Muqbil I., Aboukameel A., Senapedis W., Baloglu E., Landesman Y., Philip P.A., Azmi A.S. (2019). Targeting Rho GTPase effector p21 activated kinase 4 (PAK4) suppresses p-Bad-microRNA drug resistance axis leading to inhibition of pancreatic ductal adenocarcinoma proliferation. Small GTPases.

[8] King H., Thillai K., Whale A., Arumugam P., Eldaly H., Kocher H.M., Wells C.M. (2017). PAK4 interacts with p85 alpha: Implications for pancreatic cancer cell migration. Sci. Rep..

[9] Moon S.U., Kim J.W., Sung J.H., Kang M.H., Kim S.H., Chang H., Lee J.O., Kim Y.J., Lee K.W., Kim J.H. (2015). p21-Activated Kinase 4 (PAK4) as a Predictive Marker of Gemcitabine Sensitivity in Pancreatic Cancer Cell Lines. Cancer Res. Treat..

[10] Tyagi N., Marimuthu S., Bhardwaj A., Deshmukh S.K., Srivastava S.K., Singh A.P., McClellan S., Carter J.E., Singh S. (2016). p-21 activated kinase 4 (PAK4) maintains stem cell-like phenotypes in pancreatic cancer cells through activation of STAT3 signaling. Cancer Lett..

[11] Adamska A., Elaskalani O., Emmanouilidi A., Kim M., Abdol Razak N.B., Metharom P., Falasca M. (2018). Molecular and cellular mechanisms of chemoresistance in pancreatic cancer. Adv. Biol. Regul..

[12] Abril-Rodriguez G., Torrejon D.Y., Karin D., Campbell K.M., Medina E., Saco J.D., Galvez M., Champhekar A.S., Perez-Garcilazo I., Baselga-Carretero I. (2022). Remodeling of the tumor microenvironment through PAK4 inhibition sensitizes tumors to immune checkpoint blockade. Cancer Res. Commun..

[13] Abril-Rodriguez G., Torrejon D.Y., Liu W., Zaretsky J.M., Nowicki T.S., Tsoi J., Puig-Saus C., Baselga-Carretero I., Medina E., Quist M.J. (2020). PAK4 inhibition improves PD-1 blockade immunotherapy. Nat. Cancer.

[14] Su S., You S., Wang Y., Tamukong P., Quist M.J., Grasso C.S., Kim H.L. (2023). PAK4 inhibition improves PD1 blockade immunotherapy in prostate cancer by increasing immune infiltration. Cancer Lett..

[15] Takatsuka D., Tachinami H., Suzuki N., Yamazaki M., Yonesi A., Takaichi M., Imaue S., Yamada S.I., Tanuma J.I., Noguchi M. (2024). PAK4 inhibition augments anti-tumour effect by immunomodulation in oral squamous cell carcinoma. Sci. Rep..

[16] Ma Y., Dumesny C., Dong L., Ang C.S., Asadi K., Zhan Y., Nikfarjam M., He H. (2024). Inhibition of P21-activated kinases 1 and 4 synergistically suppresses the growth of pancreatic cancer by stimulating anti-tumour immunity. Cell Commun. Signal.

[17] Ma W., Wang Y., Zhang R., Yang F., Zhang D., Huang M., Zhang L., Dorsey J.F., Binder Z.A., O’Rourke D.M. (2021). Targeting PAK4 to reprogram the vascular microenvironment and improve CAR-T immunotherapy for glioblastoma. Nature Cancer.

[18] Luo X., Qiu Y., Fitzsimonds Z.R., Wang Q., Chen Q., Lei Y.L. (2024). Immune escape of head and neck cancer mediated by the impaired MHC-I antigen presentation pathway. Oncogene.

[19] Yamamoto K., Venida A., Yano J., Biancur D.E., Kakiuchi M., Gupta S., Sohn A.S.W., Mukhopadhyay S., Lin E.Y., Parker S.J. (2020). Autophagy promotes immune evasion of pancreatic cancer by degrading MHC-I. Nature.

[20] Mauthe M., Orhon I., Rocchi C., Zhou X., Luhr M., Hijlkema K.J., Coppes R.P., Engedal N., Mari M., Reggiori F. (2018). Chloroquine inhibits autophagic flux by decreasing autophagosome-lysosome fusion. Autophagy.

[21] Li Q., Wang S.J., Wang W.J., Ye Y.C., Ling Y.Q., Dai Y.F. (2023). PAK4-relevant proliferation reduced by cell autophagy via p53/mTOR/p-AKT signaling. Transl. Cancer Res..

[22] Ma Y., Nikfarjam M., He H. (2022). The trilogy of P21 activated kinase, autophagy and immune evasion in pancreatic ductal adenocarcinoma. Cancer Lett..

[23] He H., Dumesny C., Ang C.S., Dong L., Ma Y., Zeng J., Nikfarjam M. (2021). A novel PAK4 inhibitor suppresses pancreatic cancer growth and enhances the inhibitory effect of gemcitabine. Transl. Oncol..

[24] Gewirtz D.A. (2014). The four faces of autophagy: Implications for cancer therapy. Cancer Res..

[25] Dong L., Vaux D.L. (2020). Glucocorticoids can induce BIM to trigger apoptosis in the absence of BAX and BAK1. Cell Death Dis..

[26] Rueden C.T., Schindelin J., Hiner M.C., DeZonia B.E., Walter A.E., Arena E.T., Eliceiri K.W. (2017). ImageJ2: ImageJ for the next generation of scientific image data. BMC Bioinform..

[27] Wu T., Hu E., Xu S., Chen M., Guo P., Dai Z., Feng T., Zhou L., Tang W., Zhan L. (2021). clusterProfiler 4.0: A universal enrichment tool for interpreting omics data. Innovation.

[28] Szklarczyk D., Kirsch R., Koutrouli M., Nastou K., Mehryary F., Hachilif R., Gable A.L., Fang T., Doncheva N.T., Pyysalo S. (2023). The STRING database in 2023: Protein-protein association networks and functional enrichment analyses for any sequenced genome of interest. Nucleic Acids Res..

[29] Team R.C. (2021). R: A Language and Environment for Statistical Computing.

[30] Doncheva N.T., Morris J.H., Gorodkin J., Jensen L.J. (2019). Cytoscape StringApp: Network Analysis and Visualization of Proteomics Data. J. Proteome Res..

[31] Shannon P., Markiel A., Ozier O., Baliga N.S., Wang J.T., Ramage D., Amin N., Schwikowski B., Ideker T. (2003). Cytoscape: A software environment for integrated models of biomolecular interaction networks. Genome Res..

[32] Stifter K., Krieger J., Ruths L., Gout J., Mulaw M., Lechel A., Kleger A., Seufferlein T., Wagner M., Schirmbeck R. (2020). IFN-gamma treatment protocol for MHC-I(lo)/PD-L1(+) pancreatic tumor cells selectively restores their TAP-mediated presentation competence and CD8 T-cell priming potential. J. Immunother. Cancer.

[33] Klionsky D.J., Abdel-Aziz A.K., Abdelfatah S., Abdellatif M., Abdoli A., Abel S., Abeliovich H., Abildgaard M.H., Abudu Y.P., Acevedo-Arozena A. (2021). Guidelines for the use and interpretation of assays for monitoring autophagy (4th edition). Autophagy.

[34] Murugan S., Amaravadi R.K. (2016). Methods for Studying Autophagy Within the Tumor Microenvironment. Adv. Exp. Med. Biol..

[35] Munafo D.B., Colombo M.I. (2001). A novel assay to study autophagy: Regulation of autophagosome vacuole size by amino acid deprivation. J. Cell Sci..

[36] Das S., Shukla N., Singh S.S., Kushwaha S., Shrivastava R. (2021). Mechanism of interaction between autophagy and apoptosis in cancer. Apoptosis.

[37] La Gruta N.L., Gras S., Daley S.R., Thomas P.G., Rossjohn J. (2018). Understanding the drivers of MHC restriction of T cell receptors. Nat. Rev. Immunol..

[38] Neefjes J., Jongsma M.L., Paul P., Bakke O. (2011). Towards a systems understanding of MHC class I and MHC class II antigen presentation. Nat. Rev. Immunol..

[39] Baleeiro R.B., Bouwens C.J., Liu P., Di Gioia C., Dunmall L.S.C., Nagano A., Gangeswaran R., Chelala C., Kocher H.M., Lemoine N.R. (2022). MHC class II molecules on pancreatic cancer cells indicate a potential for neo-antigen-based immunotherapy. Oncoimmunology.

[40] Hamarsheh S., Gross O., Brummer T., Zeiser R. (2020). Immune modulatory effects of oncogenic KRAS in cancer. Nat. Commun..

[41] Han Y., Liu D., Li L. (2020). PD-1/PD-L1 pathway: Current researches in cancer. Am. J. Cancer Res..

[42] Gradiz R., Silva H.C., Carvalho L., Botelho M.F., Mota-Pinto A. (2016). MIA PaCa-2 and PANC-1—Pancreas ductal adenocarcinoma cell lines with neuroendocrine differentiation and somatostatin receptors. Sci. Rep..

[43] Xu S., Ma B., Jian Y., Yao C., Wang Z., Fan Y., Ma J., Chen Y., Feng X., An J. (2024). Development of a PAK4-targeting PROTAC for renal carcinoma therapy: Concurrent inhibition of cancer cell proliferation and enhancement of immune cell response. EBioMedicine.

