# Supplementary material for: Knockout of p21-Activated Kinase 4 Stimulates MHC I Expression of Pancreatic Cancer Cells via an Autophagy-Independent Pathway
Source: Cancers (Basel). 2025 Feb 3;17(3):511. doi: 10.3390/cancers17030511 (PMC11817421; doi:10.3390/cancers17030511)
Supplement: Supplementary file 1 [file cancers-17-00511-s001.zip › cancers-3413536-supplementary.pdf]

Supplementary Materials

Knockout of p21-Activated Kinase 4 Stimulates MHC I Expression of Pancreatic Cancer Cells via an Autophagy-Independent Pathway

Yi Ma, Chelsea Dumesny, Li Dong, Ching-Seng Ang, Mehrdad Nikfarjam and Hong He

Supplementary Method

Analysis of PAK4 knockout CRISPR event

Genomic DNA from PAK4 wild-type (WT) and knockout (KO) cell lines was extracted and purified using the Wizard® Genomic DNA Purification Kit (Promega, Madison, Wisconsin, USA) according to the manufacturer's instructions. Target regions were PCR-amplified using primers listed in Supplementary Table 1 with Q5® High-Fidelity DNA Polymerase (NEB, Ipswich, MA, USA), according to the manufacturer's protocol. PCR products were electrophoresed on a 1X TAE gel and purified using the Wizard® SV Gel and PCR CleanUp System (Promega, Madison, Wisconsin, USA). The DNAs from the WT and KO lines were subjected to Sanger sequencing using the primers listed in Supplementary Table 1 and were detected by AGRF Company. Sequencing results were aligned, and the insertion and deletion (indel) were analysed using the ApE software (https://www.bioinformatics.nl/molbi/ApE/).

a

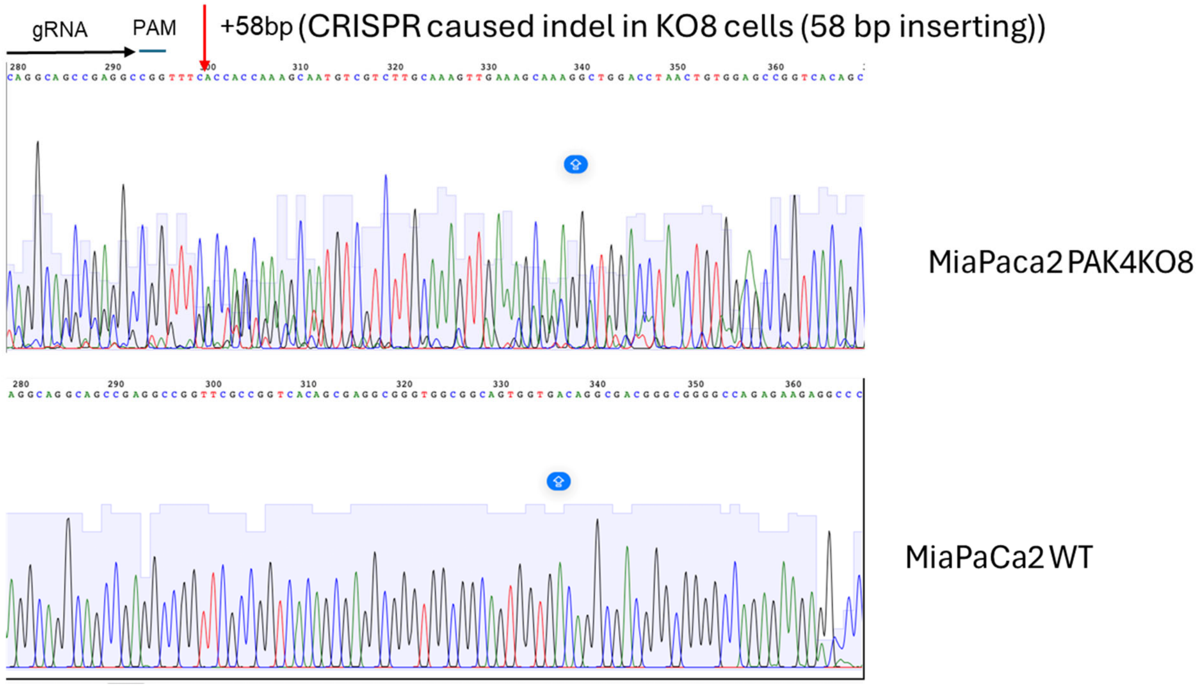

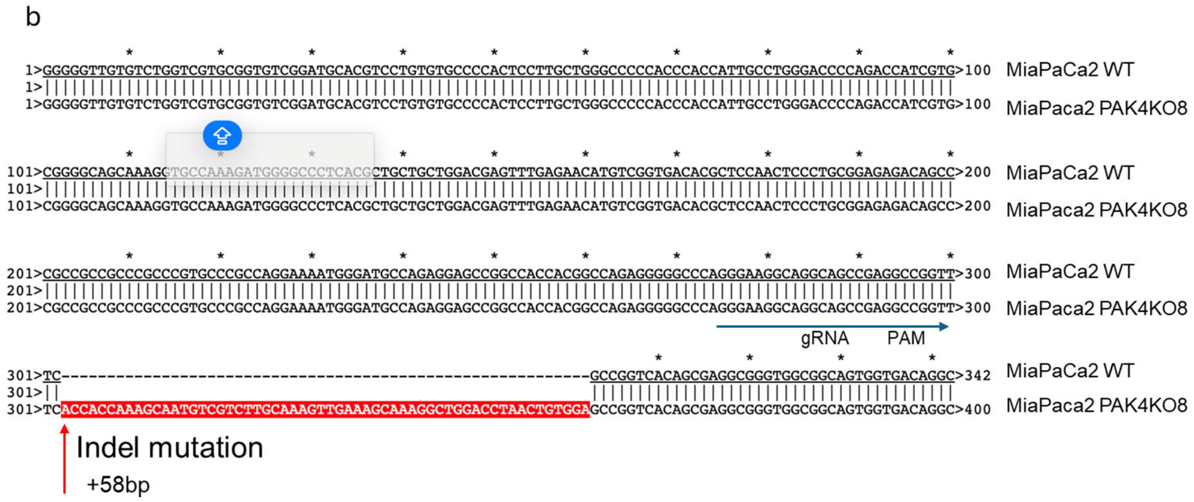

Figure S1. Sequence analysis of PAK4KO CRISPR events. The genomic DNAs of wild-type (WT) and PAK4 knockout (KO) cells were extracted, and the inserts were sequenced as described in the Supplementary Method. a) sequences for WT and KO MiaPaCa-2. PAM sequence, gRNA binding and Cas9 cut site were marked. The arrow indicated a 58 bp insert for CRISPR-caused insertion and deletion (indel). b) The sequence of WT was aligned with the sequence of PAK4KO. The red sequences showed the indel of 58 nucleotide insertions.

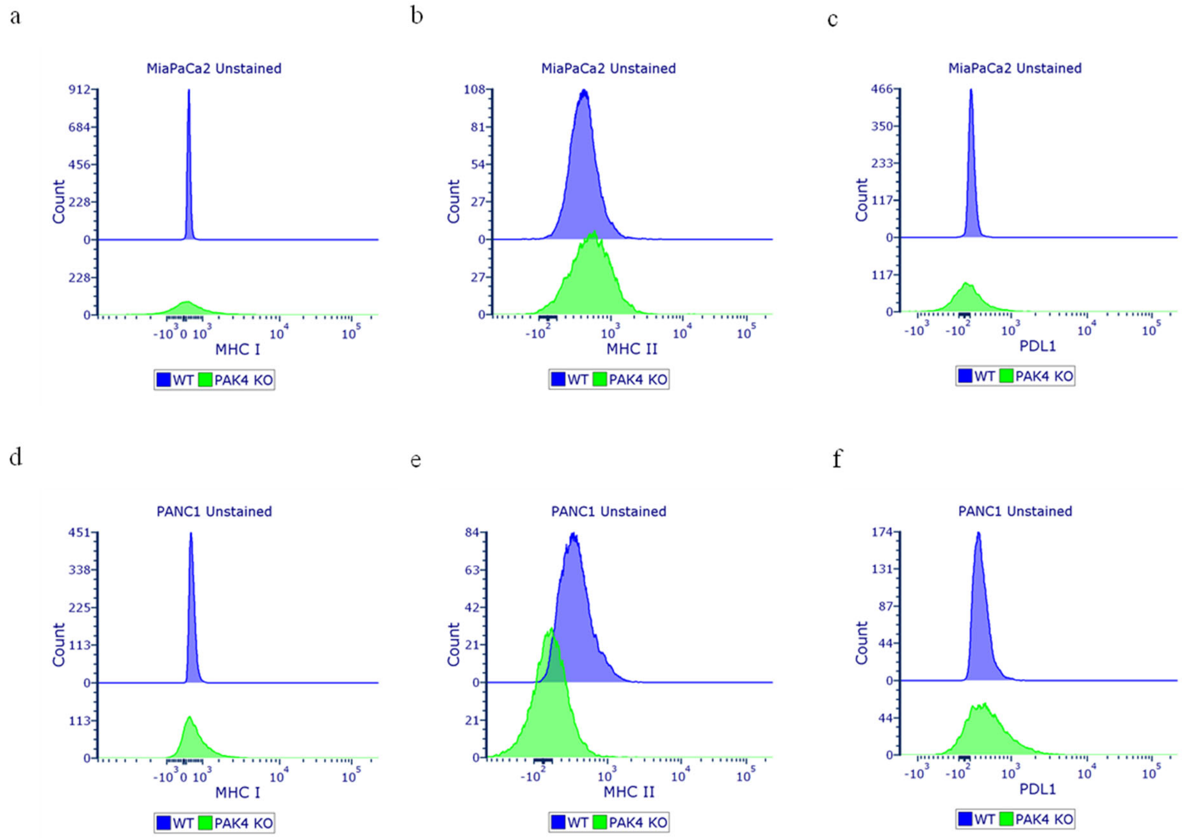

Figure S2. Unstained WT and PAK4 KO controls for cell surface MHC I, MHC II and PDL1 FACS analysis after compensation. (**a-c**) Unstained controls of WT and PAK4 KO MiaPaCa-2 cells for MHC I, MHC II and PDL1 respectively. (**d-f)** Unstained controls of WT and PAK4 KO PANC-1 cells for MHC I, MHC II and PDL1 respectively.

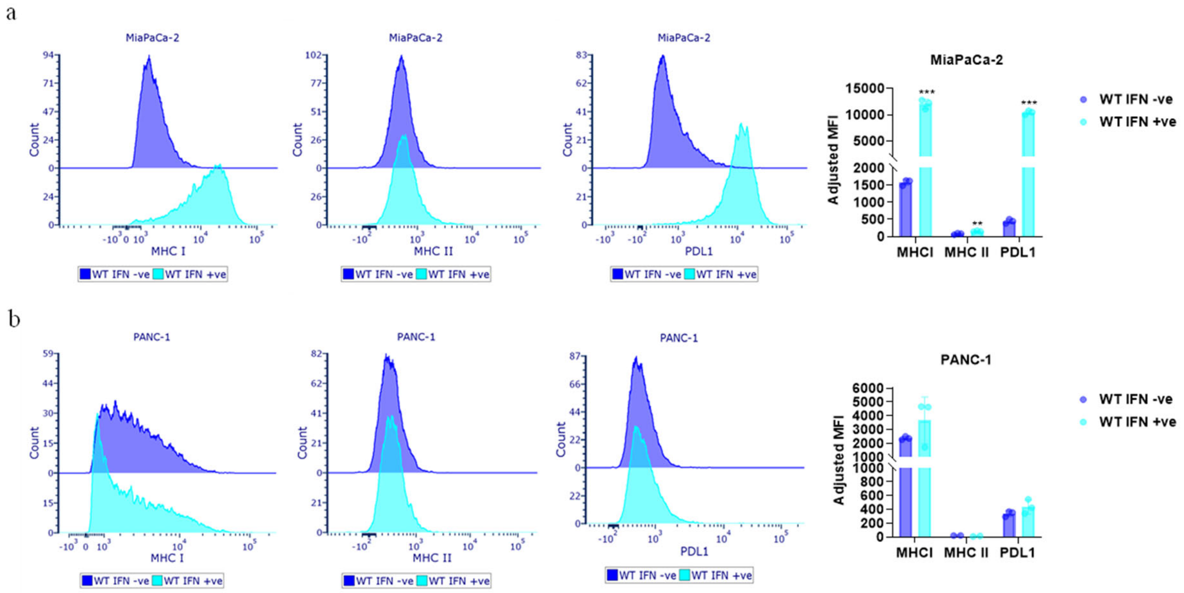

Figure S3. IFN-g induced MHC I and PDL1 expression on MiaPaCa-2 and PANC-1 cell lines. The cell surface expression of MHC I, MHC II and PDL1 were determined by FACS analysis. The expression of MHC I, MHC II and PDL1 by MiaPaCa-2 (a) but not PANC-1 (b**)** were increased by IFG. **p<0.01, ***p<0.001. All comparisons were made against WT unless otherwise indicated.

a

b

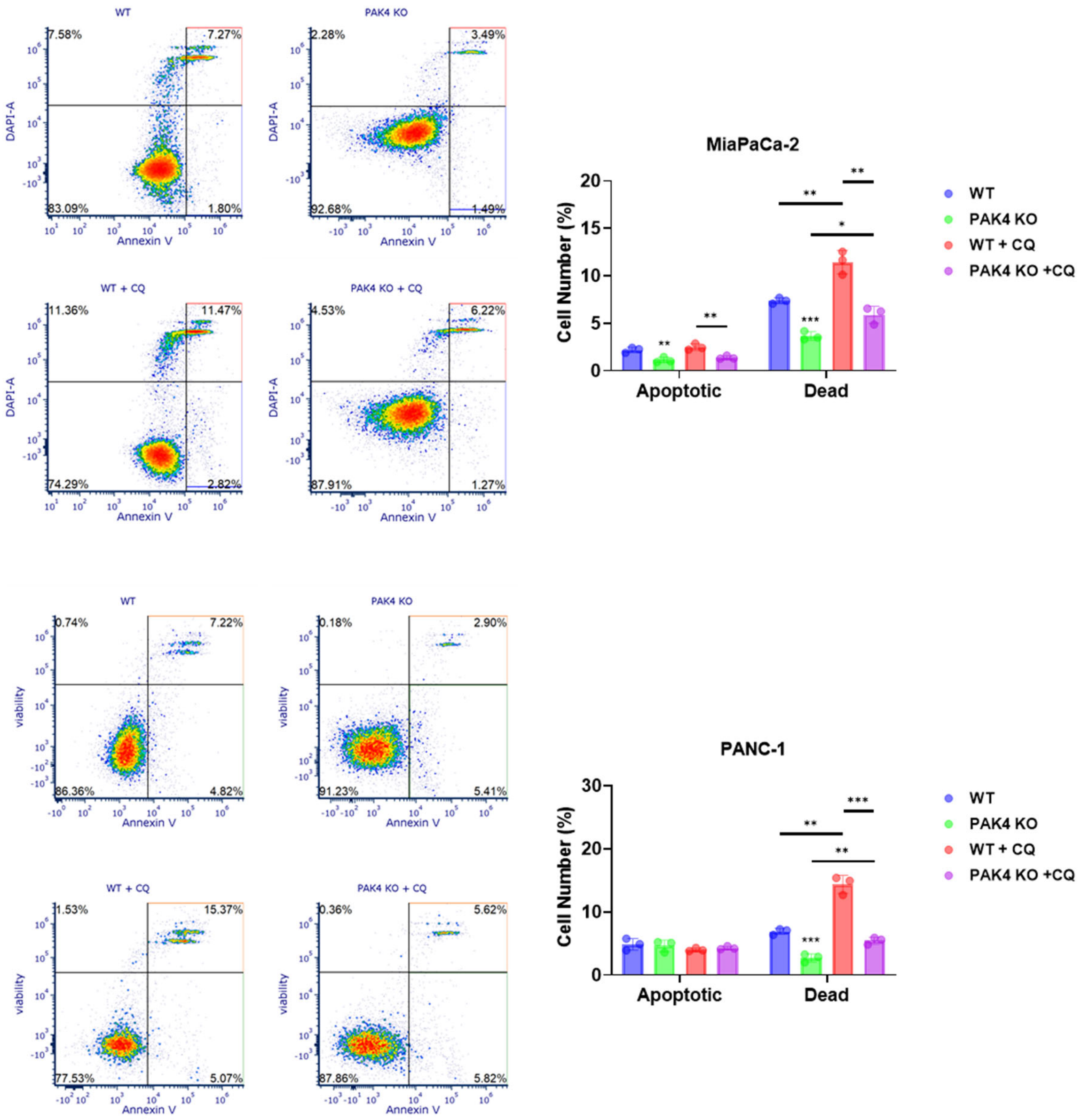

Figure S4. Inhibition of autophagy by chloroquine did not affect the effect of PAK4 knockout on apoptosis and cell death. **(a)** FACS analysis of apoptosis and cell death in wild type (WT) and PAK4 knockout (KO) MiaPaCa-2 cells treated with or without chloroquine. (**b)** FACS analysis of apoptosis and cell death in WT and PAK4 KO PANC-1 cells treated with or without chloroquine. *p<0.05, **p<0.01, ***p<0.001. All comparisons were made against WT unless otherwise indicated.

Table S1. Primers for PAK4 CRISPR events.

	**Pair 1**	**Pair 2**
Forward	TGGTCCCGGTGTAAGATGAG	TGCGTCTCTGTCTTGTCTCT
Reverse	CCATGTCAGTGAGGAGGTGT	AGACTTGGGCCTCTTCTCTG
PCR product	433bp	398 bp

Table S2. Buffers used in the method.

Buffers	Content
RIPA cell lysis buffer	25mM Tris HCL, 150mM NaCl, 1% Triton X-100, 1% Na deoxycholate, 0.5% SDS, 1mM EGTA, PH 8
ONYX cell lysis buffer	20mM Tris, 135mM NaCl, 1.5mM $MgCl_2$, 1mM EDTA, 10% Glycerol, 1% Triton X-100, PH7.5
FACS buffer	1X PBS, 2%FBS, 0.05% Sodium Azide

Table S3. Primary antibodies for immunoblot.

Protein target	Dilution	Cat. number	Company
PAK4	1:1000	PA5-15120	Invitrogen
LC3B	1:1000	3868S	Cell Signaling & Technology
ATG5	1:1000	PA1-46178	Invitrogen
Beclin 1	1:1000	PA1-16875	Invitrogen
SQSTM1	1:1000	ab91526	Abcam
BCL2	1:1000	ab182858	Abcam
GAPDH	1:10000	2118S	Cell Signaling & Technology

Table S4. Primary antibodies for flow cytometry.

Protein target	Fluorophore	Dilution	Cat. number	Company
HLA-ABC (MHC I)	PE	1:200	130-120-141	Miltenyi Biotec
HLA-DR (MHC II)	APC	1:200	307610	BioLegend
CD274 (PDL1)	PE-Cy7	1:200	558017	BD Pharmingen
